# Lab-in-a-Droplet: Utilizing Acoustic Levitation for Miniaturized Sample Preparation and LC-MS/MS, Exemplified by Measurement of Vitamin D in Serum

**DOI:** 10.1021/acs.analchem.6c03465

**Published:** 2026-07-22

**Authors:** Geron Ratzek, Dietrich A. Volmer

**Affiliations:** Department of Chemistry, 9373 Humboldt-Universität zu Berlin, Brook-Taylor-Straße 2, Berlin 12489, Germany

## Abstract

Conventional sample preparation of biological matrices typically requires sample sizes ranging from micro- to milliliters. For difficult-to-obtain samples such as newborn blood or breast milk, collecting sufficient volumes can be challenging, thereby limiting the application of established *in vitro* assays. Performing the entire sample preparation step within a single levitated droplet (*in stillo*) of only a few microliters of the sample can address this challenge by reducing the required sample size while shortening sample preparation time and promoting green chemistry principles. In this proof-of-concept study, we demonstrate the feasibility of acoustic levitation as a miniaturization strategy, exemplified by the measurement of 25-hydroxyvitamin D in human serum samples. Preliminary experiments demonstrated that contactless handling of vitamin D samples via acoustic levitation was feasible, including liquid–liquid extractions with excellent recoveries. A [4 + 2]-Diels–Alder derivatization reaction for sensitivity improvement can be conducted *in stillo* within the levitated droplet. A direct comparison between *in stillo* and *in vitro* derivatization suggested a 60-fold increase in reaction rate and a corresponding reduction in analysis time. We successfully extracted and derivatized 25-hydroxyvitamin D from human serum under acoustic levitation conditions and subsequently detected the analyte with LC-MS/MS. Side-by-side evaluation indicated a 15.4% deviation between *in stillo* and *in vitro* results, with slightly lower response factors for the *in stillo* workflow, readily demonstrating the general feasibility of the miniaturization approach. Further optimization of droplet handling, including the injection and ejection processes, will undoubtedly improve the analytical figures of merit, indicating promising performance in future matrix and validation studies.

## Graphical abstract

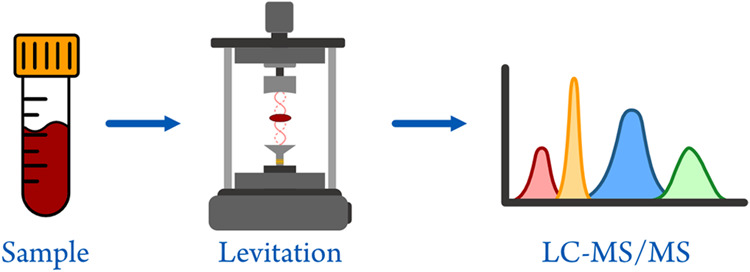

Blood remains the most important sample matrix for clinical assessment of numerous biomarkers.^1^ However, sampling is invasive and causes discomfort for patients, especially when large volumes or frequent sampling are required.^2^ Several demographic groups are more prone to complications during sampling, such as older adults, neonates, or anemic patients.^3^ Since blood is a complex matrix, cleanup and preconcentration steps are necessary before analysis. Protein precipitation and liquid–liquid extractions are commonly utilized for blood samples.^4^ Nevertheless, large sample volumes (e.g., μL to mL) are often required for efficient and reliable sample preparation.^4–6^ Such large sample sizes may not be available due to the invasive nature of the sampling and possible interference with patient conditions. According to Lippi et al., 11% of analytical errors arise from insufficient sample volume during the preanalytical steps.^6^ One way to address these issues is the reduction of the required sample volume through miniaturized sample preparation, which would ultimately decrease sampling invasiveness. Miniaturized sample preparation is particularly valuable in areas such as clinical research, drug discovery, and personalized medicine, to enable high-throughput screening and analysis of limited sample volumes.^7^ Additionally, downsizing the experimental sample preparation routine follows the principles of green chemistry, as less solvent and analytical reagents are required and less (toxic) waste is produced.^8^ In addition to well-established microfluidics approaches,^9–12^ levitation of sample droplets has emerged as an alternative sample preparation strategy. There are several means of achieving sample levitation, in particular optical,^13^ electrostatic,^14^ magnetic,^15^ and acoustic levitation.^16^ Acoustic levitation was of particular interest for this study because it exhibits minimal sample degradation and unwanted reactions in comparison to other levitation techniques.^17,18^

Acoustic levitation is slowly gaining traction in the chemical sciences due to its unique and advantageous properties.^16,19,20^ By generating a standing wave at ultrasonic frequencies, periodic high- and low-pressure nodal points form, allowing contactless suspension of liquid (nL to μL) and solid samples in midair (Figure S1 provides an illustrative schematic of an acoustic levitator; Supporting Information).^16^ This approach has several advantages over other miniaturization approaches. The contactless approach reduces the risk of possible chemical and thermal contamination, which can be introduced through container walls.^21^ Additionally, absence of container walls reduces unwanted noise signals during measurement (e.g., scattering in UV experiments and XRD).^21^ Of particular interest are additional phenomena in a single levitating droplet (*in stillo*)^22^, in particular, laminar flows (acoustic streaming).^23^ Acoustic streaming significantly enhances homogenization and reaction kinetics,^24^ enhanced by the droplet’s large surface-to-volume ratio.^25,26^ Given that a wide variety of liquids and materials can be readily levitated,^16^ it is conceivable that numerous *in vitro* reactions or analytical assays can be adapted to *in stillo* workflows.

It has long been established that sufficient vitamin D levels contribute to the healthy regulation of calcium and phosphate, thereby supporting the development of healthy bones and muscles.^27,28^ Recent studies have suggested that the bioactive metabolite 1,25-dihydroxyvitamin D (1,25­(OH)_2_D)^29^ contributes to several further key functions in the human body through vitamin D-regulated transcription of numerous proteins.^30^ Therefore, an underlying correlation between insufficient vitamin D levels and several chronic diseases was observed. Vitamin D deficiency has also been linked to increased risk of cancer,^31^ cardiovascular diseases,^32,33^ autoimmune diseases such as multiple sclerosis,^28,34^ and neurological disorders such as depression,^35^ dementia^36^ and Parkinson’s disease.^37^ Health concerns are increasing, as a significant proportion of the population is deficient in vitamin D.^38,39^ According to Cashman et al., 13.0% of Europeans exhibit insufficient 25-hydroxyvitamin D (25­(OH)­D) levels (<30 nmol/L).^40^ Depending on the definition of deficiency, this percentage increases to values up to 40.4% (<50 nmol/L).^40^ Liquid chromatography-tandem mass spectrometry (LC-MS/MS) is considered the “gold standard” method for the clinical assessment of vitamin D levels in human blood.^41,42^

What prompted the current study was the almost complete lack of experimental investigations on sample preparation aspects within an acoustic levitation system. Most studies demonstrate analytical applications of acoustic levitation within a single droplet using previously prepared samples.^19,20,43–46^ While virtually every analytical study on acoustic levitation describes the advantages of the containerless environment, in particular the minimized risk of contamination during analysis, samples have virtually always been cleaned up before acoustic levitation; that is, target analytes in solvent, with little or no interfering (biological) matrix components. Obviously, samples have to be cleaned up before acoustic levitation-MS, which potentially introduces additional interferences and generally makes the claim of limited contamination risk somewhat problematic if sample preparation is performed outside the acoustic levitator.

For integrating acoustic levitation into more complex workflows, comprising sample introduction, sample manipulation (including cleanup + derivatization), and analyte detection, contactless manipulation of the samples inside the acoustic field has to be developed. Recent studies have shown that solvent coalescence^47^ and mixing^48^ are readily possible, suggesting the possibility for more complex workflows, allowing miniaturization of the sample preparation workflow of very small sample sizes, while simultaneously adhering to green chemistry principles.

The objective of this proof-of-concept study is the development of a lab-in-a-droplet analytical workflow, integrating sample handling and preparation, for biological samples within the confined space of an acoustically levitated single microdroplet and subsequent analysis by mass spectrometry. The approach described here is different from microfluidics methods, which utilize much smaller droplet sizes (fL to nL). Here, we aim to reproduce an established analytical benchtop workflow within a single microdroplet (*in stillo)* of a few μL. This will enable measurements with significantly reduced sample and reagent consumption in contrast to regular vessels as well as minimized risk of contamination or adsorption to container walls during analysis. In comparison to microvessels, the unique properties of microdroplets also provide conditions that allow important processes, such as mixing or chemical reactions, to be performed at highly accelerated rates in comparison to *in vitro* reactions in regular vessels allowing for increased efficiency of each sample preparation step, while ensuring sample integrity of thermally labile or light sensitive samples.

To our knowledge, no previous work has described the application of acoustic levitation for complex sample preparation steps of biological samples. In this study, we will implement an analytical workflow for the analysis of vitamin D samples in human blood serum as proof-of-concept and compare the obtained data to an established and validated in-house LC-MS/MS assay for 25-hydroxyvitamin D (25­(OH)­D). 25­(OH)­D serves as a representative model analyte, as its lipophilicity and predominant protein-binding present demanding conditions for the sample preparation.

## Methods

### Chemicals and Materials

All chemicals and pooled human serum were obtained from commercial suppliers (detailed sources are provided in the experimental section of the Supporting Information).

### Acoustic Levitation

Acoustic levitation was performed using a commercial acoustic levitator (Dante Dynamics, Skovlunde, Denmark). The transducer emitted an ultrasonic wave at 58 kHz and operated in the HF power range of 0.65–5 W. The distance between the transducer and reflector was set to 2.58 cm and was only slightly adjusted (0.02 cm) to bring the droplet in and out of oscillation during extractions. Spatial manipulations and displacements were achieved by rapid alternation of the power output (ΔHF ≈ 0.4–0.7 W). Solvents, standards, and samples were injected into the central acoustic pressure node using 5 or 10 μL gastight Hamilton (Bonaduz, Switzerland) syringes. Optical images of acoustically suspended droplets were magnified with a Pentax VM 6 × 21 macro lens (Pentax, Rungis, France). Levitation and sample preparation were performed under ambient conditions at room temperature (20–25 °C).

### Liquid Chromatography-Tandem Mass Spectrometry

Liquid chromatography was performed on an Agilent (Wilmington, DE, USA) 1290 Infinity II LC system. A Kinetex (Phenomenex, Torrance, CA, USA) 2.6 μm C-18 100 Å (100 × 2.1 mm) column, was used for chromatographic separation. The mobile phase consisted of water (+0.1% FA) and methanol (+0.1% FA), which were degassed for 15 min before analysis. A linear gradient with an increase from 30 to 100% of the organic phase within 13 min was chosen. Subsequently, isocratic elution at 100% methanol was performed before returning to the initial conditions. The flow rate was set to 0.4 mL/min, and the column temperature to 30 °C. The LC system was coupled to a QTRAP 6500+ triple quadrupole linear ion trap mass spectrometer (Sciex, Concord, ON, Canada). Ionization in positive ion mode was conducted with a Turbo-V electrospray ionization (ESI) source and data acquired in multiple reaction monitoring (MRM) and full-scan modes using Analyst software (Sciex) version 1.7. Integration and quantification were performed using MultiQuant (Sciex) version 3.0.3.

### Vitamin D Sample Preparation

Five μL of the untreated serum sample was injected into the centric nodal point of the acoustic field. After 20 s of equilibration time, 5 μL of dry acetonitrile was added to the levitating sample, triggering immediate precipitation of the proteins. The acoustic field strength was slowly adjusted (ΔHF = 0.2 W) to agglomerate the precipitate at the bottom of the droplet. After ca. 30 s equilibration time, the droplet was left to evaporate for 3 min under ambient conditions. Subsequently, 5 μL of ethyl acetate was injected into an adjacent nodal point and spatially moved toward the sample node by rapidly alternating the acoustic pressure. Extraction was carried out by inducing oscillation with subsequent mixing of the two droplets for 30 s. Subsequently, rapid modulation of the acoustic field pressure induced spatial separation of the two phases into adjacent nodes. The organic phase was manually collected and an additional 5 μL of ethyl acetate was added to the sample droplet. The organic phases were then combined and transferred to the central nodal point. The combined organic phase was allowed to evaporate to near dryness under ambient conditions for approximately 3 min. Subsequently, 10 μL of PTAD solution (200 ng/mL, in ACN) was added to the organic phase and the reaction was allowed to proceed for 3 min. The sample was left in a levitated state for an additional 2 min to facilitate evaporation, then quenched with methanol. The sample was manually collected and diluted with MeOH/H_2_O (85/15 v/v) to a final volume of 100 μL. The extract was then measured by LC-MS/MS. Full experimental details and calibration data (Figures S2 and S3) are provided in the Supporting Information.

## Results and Discussion

The primary aim of the present study was to adapt an established *in vitro* sample preparation protocol to the “lab-in-droplet” environment, in which all analytical steps are performed within a single microdroplet of only a few microliters. Achieving this required not only scaling down the workflow but also required the development of technical solutions specific to acoustic levitation, to enable operations that are straightforward under conventional laboratory conditions. These include sample handling by pipetting, mixing, liquid–liquid extraction, including phase separation and chemical reactions, all of which are typically carried out in suitable vessels in the macroscopic *in vitro* environment.

### Spatial Manipulation of Droplets and Liquid–Liquid Extraction

To perform liquid–liquid extraction under acoustic levitation conditions, we placed the sample and the extraction solvent droplet into adjacent low-pressure nodes with no liquid contact. The two immiscible phases were then merged, followed by coalescence and mixing. Phase separation was achieved by moving the two phases to their original pressure nodes. Table S1 provides additional examples of immiscible solvents for droplet levitation.

All individual steps for this operation were performed along the *z*-axis of a single-axis levitator. By rapidly alternating the power output and thus the acoustic pressure (ΔHF ≈ 0.4–0.7 W), the acoustic field was rapidly destabilized and stabilized, leading to spatial displacement of one or more droplets within the acoustic field (Figure 1). This not only allowed for the liquid–liquid extraction using immiscible solvent droplets, but also enables contactless merging of miscible solvents, which can be utilized, e.g., for dilution steps. When two immiscible solvents (e.g., water and 1-octanol) are injected into adjacent nodal points, they can be merged by rapid field modulations, which also results in intensive mixing of the two droplets (see, Supporting Information Figure S4A). Careful control of acoustic pressure is essential for this step, as otherwise the merging process is too forceful and unwanted air pockets or phase partitioning are sometimes introduced. To avoid this, the acoustic field pressure was carefully modulated, leading to agglomeration of the air cavities on the surface and pronounced phase separation. To improve repeatability, other parameters besides the HF parameters, such as frequency, distance, injection position and axial alignment, were kept constant during field modulation. Additional examples for spatial manipulation and merging of droplets are shown in Figures S5–S9 (Supporting Information).

**Figure 1:**
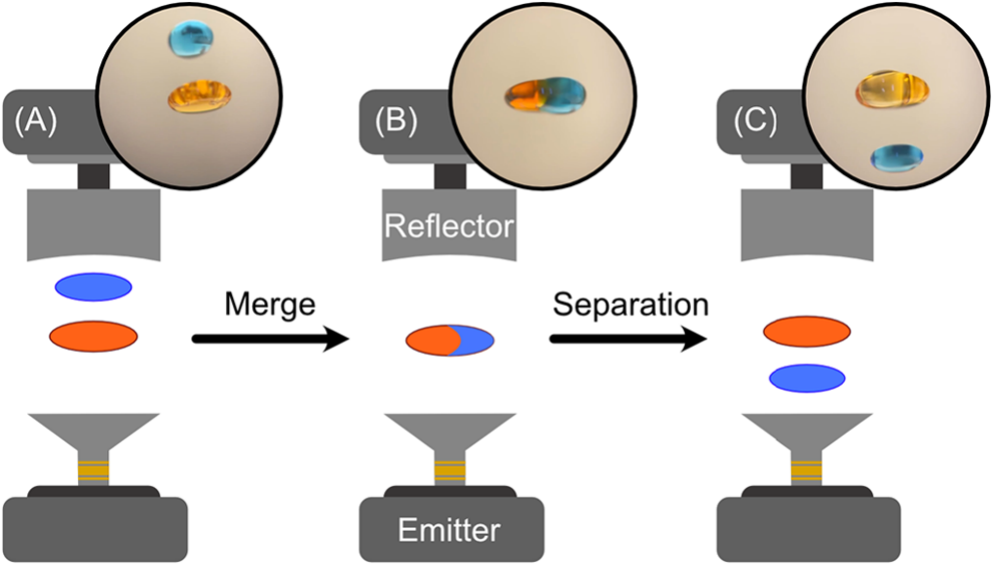
Schematic representation of the proposed *in stillo* workflow, with (A) initial sample (water, dyed blue with indigo carmine) and solvent (1-octanol, dyed orange with methyl red), (B) merged droplets and (C) separated droplets.

The two merged-phase droplets were separated into individual low-pressure nodes by rapid, successive changes in acoustic sound pressure (Figure 1B; Supporting Information, Figure S4B). To achieve clean phase separation, the levitation conditions had to be carefully controlled with the acoustic levitator used in this study, which unfortunately did not allow full computer control of the acoustic pressures. Therefore, separation patterns other than complete separation were observed in ca. 20% of all experiments, where small amounts (≈0.5 μL) of residual second phase remained attached to the isolated droplet, appearing as dispersed droplets on the surface of the main phase (Supporting Information, Figure S4C). Gradual modulation of the acoustic pressure field enabled the separated fractions to coalesce on one side of the droplet, thereby enhancing separation efficiency. Future implementation of computer-controlled levitator operation is expected to provide more precise control of the separation process. Only infrequently (<5% of experiments), the lower-density solvent underwent droplet separation in a manner where a substantial portion (0–2.5 μL) remained associated with the primary droplet, while a smaller fraction was displaced into an adjacent pressure node (see Supporting Information, Figure S4C).

This behavior was likely attributable to exceeding the upper pressure threshold tolerable (HF > 4.5 W) for the volatile solvent, which can induce violent atomization of the low-density phase,^49^ leading to an uncontrolled separation. For our experimental setup, the solvents for sample preparation therefore needed to be carefully chosen and evaluated, as differences in surface tension, viscosity and vapor pressure have a significant influence on the acoustic parameters. For improved phase separation without attached residual solvent to the isolated droplet, more precise sound pressure modulation is required. Full separation was achieved when specific HF values (ΔHF = 0.4–0.7 W, depending on the solvent) were rapidly alternated, therefore mimicking a computer-controlled acoustic field. Fast cycling between the HF values minimized unwanted oscillations and thus avoided fragmentation during the separation. Additionally, smaller droplets possess higher surface-to-volume ratios, allowing the surface tension to overcome the inertia of the droplet’s bulk mass, thus reducing fragmentation. In the future, we will combine automated spatial manipulation with smaller droplets (e.g., 2 μL) to further improve phase separation while minimizing sample size.

Compared to a previous study on phase partitioning of immiscible liquids in an acoustic levitator,^50^ we did not observe different phase layers. In fact, we only observed the formation of a single interfacial plane or partially dispersed phases due to vigorous merging. Since the interfacial area between the phases is small, we considered the possibility that the extraction efficiencies may be limited, especially since vigorous mixing occurred only during the initial merging. To investigate the efficiency of the extraction process further, we performed a series of *in stillo* extractions of 25-hydroxyvitamin D_3_ under different conditions (Figure 2). Our previously established validated assay for vitamin D metabolites^51^ utilized a protein precipitation step using acetonitrile, before conducting extraction into ethyl acetate. As we will also implement an *in stillo* protein precipitation step (*vide infra*), it was important to study the extraction of 25­(OH)­D_3_ from acetonitrile into ethyl acetate under acoustic levitation conditions, to assess the efficiency of the recovery of the process. For single-step extraction, the migration of the analyte was initially monitored using only the small interfacial plane without additional oscillations (vibration). The concentration in the extraction solvent gradually increased until a maximum was reached at 3 min at ≈75% transfer, followed by a sharp decline at longer extraction times (Figure 2). We attribute this decrease to either partial combustion of the droplet or to redistribution of the analyte. Figures S10–S12 (Supporting Information) provide additional examples for droplet merging, mixing and liquid–liquid extractions.

**Figure 2:**
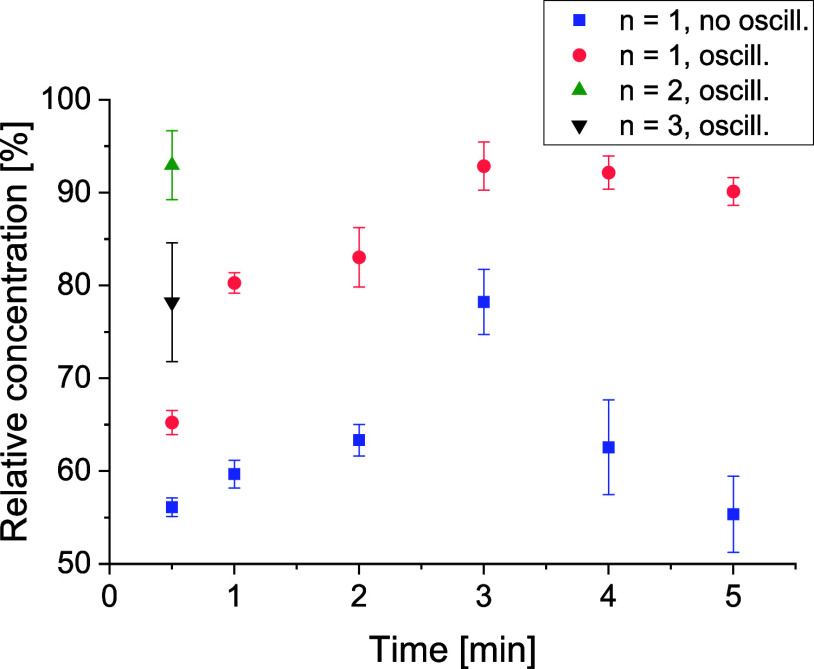
Relative concentration (=analyte recovery) of 25­(OH)­D_3_ in the ethyl acetate droplet after extraction from the acetonitrile sample droplet under acoustic levitating conditions. Data shown for single, double and triple extraction. Error bars represent the absolute data range (*n* = 2).

When additional oscillations are applied to the droplets by adjusting the distance between the transducer and reflector, the initial recovery yield substantially increased (≈95%), with the highest yields again at 3 min, but without a subsequent decrease after 3 min. This observation suggests that oscillation and acoustic streaming effects substantially aid in the extraction during liquid–liquid extraction. The oscillation disturbs the ultrasonic field and causes rapid mixing of the two phases. The mixing of two immiscible liquids (sample and extractant) is more complex than that of two miscible solvents, as there are effects from differences in density, viscosity and surface tension between the droplets, where the interfacial tension between the immiscible liquids creates boundaries at the interface between each droplet.^52^ When droplets of immiscible liquids come into contact, they may undergo coalescence due to the reduction of interfacial tension caused by the acoustic field. As a result, the immiscible liquids merge and begin to mix at the microscale within the combined droplet.^52^ We suggest that in our experiment, initially, the concentration gradient and partition coefficient aid the extraction process, but acoustic streaming effects outweigh the diffusion limit and prevent the analyte from distributing back into the sample, thereby increasing *in stillo* extraction yields. We also performed two multistep extractions (*n* = 2, 3). The efficiency of the two-step extraction exceeds that of the corresponding single-step extraction, as expected from comparable conventional *in vitro* extractions. However, performing multiple extractions did not increase the overall efficiency as much as theoretically expected, which could be due to partial combustion of the extractant droplet or losses during sample transfers. As the future complete lab-in-a-droplet method will include direct mass spectrometric analysis of the droplet (see Conclusions), we will likely implement a single-step extraction routine to preserve analyte integrity, minimize sample loss and accelerate sample throughput.

### Enhanced Chemical Derivatization in the Droplet

The *in stillo* experiments also exhibit unique reaction conditions in the confined space of the microdroplets. Previous work has shown that *in stillo* reactions exhibit a strong increase in turnover rates.^24^ Reaction confinement and its role in reaction kinetics have been discussed by Fallah-Araghi et al.^26^ The authors attributed the reaction acceleration and enhanced yields in micrometer-sized droplets to a mechanism where the reactants are adsorbed to the droplet’s surface, react, and then diffuse back into the bulk of the droplet. Another major contributing factor to the observed rate enhancements is the rapid mixing described above, which suggests that for comparable droplet volumes, mixing in the droplet far outweighs diffusion.

In this study, chemical derivatization in droplets was investigated to improve the detection sensitivity for the analytes. For vitamin D metabolites, Cookson-type reagents are commonly used to strongly increase detection sensitivity.^4,51^ Here, we studied the derivatization of 25-hydroxyvitamin D_3_ with the well-established 4-Phenyl-1,2,4-triazolin-3,5-dion (PTAD) reagent (Figure 3).

**Figure 3:**
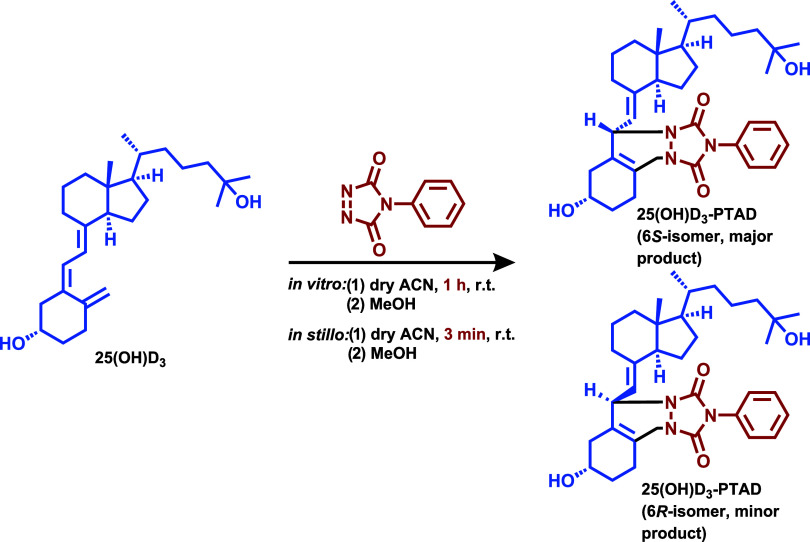
[4 + 2]-Cyclo-addition derivatization reaction of 25­(OH)­D_3_ (blue) with PTAD (red) under *in vitro* and *in stillo* conditions.

Our previously reported *in vitro* protocol for PTAD conducted the derivatization reaction in acetonitrile under ambient conditions at room temperature.^51^ A reaction time of 1 h was used, followed by quenching the reaction with methanol. Some of these conditions are challenging to implement under acoustic levitation conditions due to the uncontrolled ambient conditions during droplet suspension; e.g., moisture from the air and temperature changes would potentially have a significant influence on the reaction. To evaluate the feasibility of *in stillo* vitamin D derivatization, we compared the extent of product formation of 25­(OH)­D_3_-PTAD under *in stillo* and *in vitro* conditions (Figure 4). Under *in vitro* conditions, product formation steadily increased, with the highest yield reached after 60 min, consistent with previous data.^51^ Interestingly, *in stillo* product formation reached the same yield after less than 1 min, indicating at least a 60-fold increase in reaction rate for this specific derivatization reaction. Further product formation seemed to occur over the next several min. However, significant solvent evaporation also occurred during this extended time as seen from relative concentration values exceeding 100% after 3 min. The sigmoidal shape of the curve suggests that initial product formation was slowly superseded by evaporation, leading to sample preconcentration. After reaction times of >10 min, excessive evaporation resulted in insufficient droplet volume (≲0.5 μL), which was difficult to manually collect in our proof-of-concept experimental setup. This limitation will be eliminated in future implementations, in which the droplets will either be directly interrogated for detection, as previously shown,^19,24,53^ or transferred directly into the mass spectrometer. We suggest that this strong increase in reaction rate is caused by the strong mixing and homogenization effects from the acoustic streaming and the droplet’s high surface-to-volume ratio.^24^ The pronounced laminar flows could also increase the rate of contact between the reactants, thereby aiding product formation. Additionally, local high-concentration hot spots are avoided, which would otherwise decrease mass transfer rates. Moreover, acoustic levitation can increase the temperature of a levitated sample,^54^ which can potentially also accelerate reaction rates. These temperature changes depend on the given levitator design and the sample size and will be studied in the future

**Figure 4:**
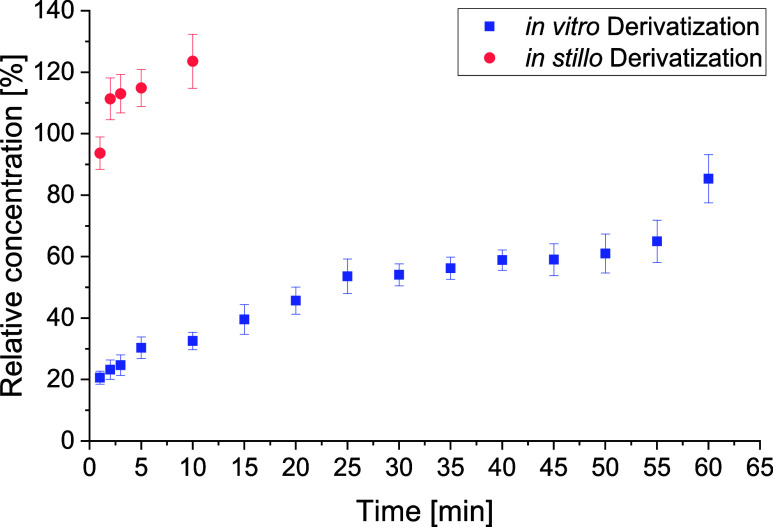
Comparison of 25­(OH)­D_3_-PTAD product formation under *in stillo* (red) and *in vitro* (blue) conditions (the reaction was performed in dry acetonitrile). Error bars represent the absolute data range (*n* = 2).

### Quantification of Vitamin D_3_ in Serum

We combined derivatization and extraction of 25­(OH)­D_3_ under acoustic levitation conditions, to demonstrate an *in stillo* sample preparation workflow with subsequent LC-MS/MS detection for direct analysis of a human serum sample. Compared to standard solutions, no differences in levitation behavior were observed. It is expected that matrix-dependent differences in serum viscosity affect the required sound pressure levels, mixing behavior and mass transfer. Upon the addition of acetonitrile, serum proteins were precipitated. The precipitate was collected at the bottom of the droplet by gradually decreasing the acoustic field pressure (Figures 5 and S13 in the Supporting Information provides additional images).

**Figure 5:**
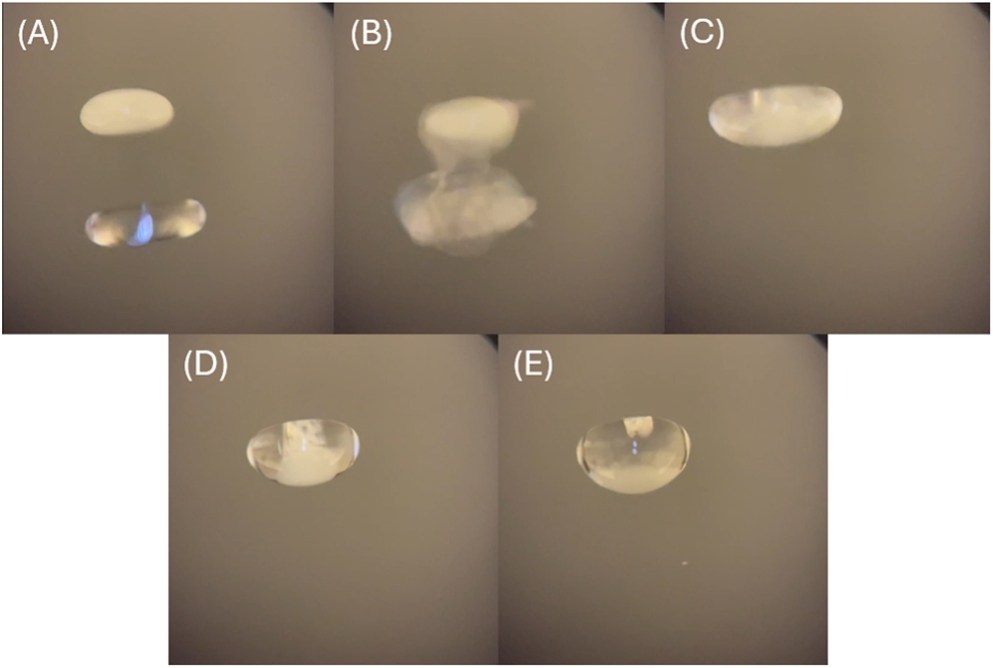
Merging of human serum (upper droplet) with acetonitrile (lower droplet) and precipitation of serum proteins: (A) droplets before merging, (B) merging process, (C) merged droplet, (D) central agglomeration of precipitate, and (E) precipitate collection at the bottom of the droplet.

During this step, a small portion of the solvent was purposely evaporated, as otherwise subsequent reagent additions to the droplet may overload the acoustic trap utilized in this study. After injecting ethyl acetate into the adjacent nodal point, the droplets were merged for liquid–liquid extraction by rapidly modulating the acoustic field pressure and droplet separation was performed, and then chemical derivatization in the extract droplet with PTAD, followed by quenching the reaction.

The feasibility of the containerless platform for (semi)­quantitative vitamin D analysis was demonstrated using a six-point calibration curve (*n* = 3), exhibiting excellent linearity (*R* ^2^ = 0.997) over the investigated concentration range of 0.5–3 ng/mL (Figure 6). Additionally, a limit of quantification (LOQ) of 0.022 ng/mL was obtained, demonstrating performance within the required operational range of the established vitamin D assay^51^ (Supporting Information, Experimental Section).

**Figure 6:**
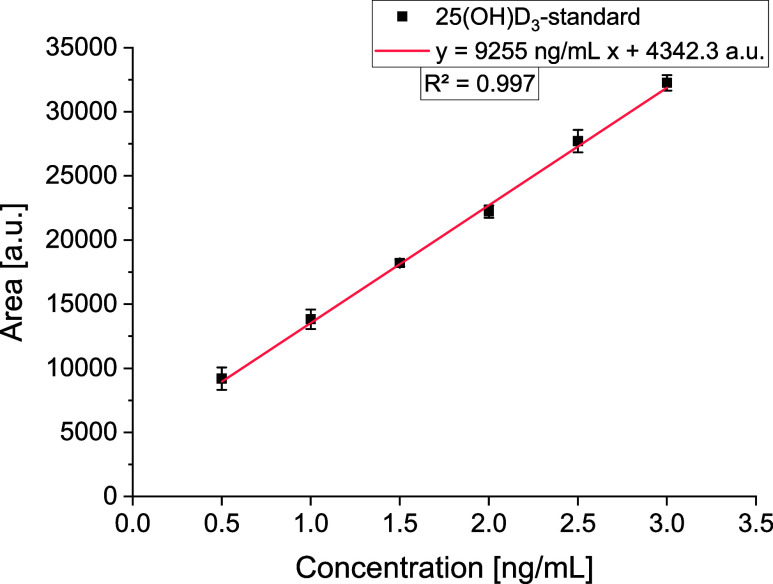
Calibration curve for measurement of 25-hydroxyvitamin D_3_ in human serum after liquid–liquid extraction using acoustic levitation. Error bars represent the standard deviation (*n* = 3).

A comparison of *in stillo* and *in vitro* full scan LC-MS chromatograms revealed few differences in the number of coextracted components from serum, where the *in vitro* extraction seems to extract more components in comparison to the *in stillo* extraction (Figures S14 and S15, Supporting Information), but there are also unique extracted compounds in both the *in vitro* and the *in stillo* extracts. These differential effects are probably due to different extraction efficiencies caused by changes in phase boundaries during liquid–liquid extraction for different matrix components in complex matrices. Extraction efficiency may also be affected by serum surfactants, which migrate toward the droplet surface, as discussed by Fallah-Araghi et al.^26^ We will address the extraction of a broader range of compounds from complex samples using the described *in stillo* routine in the future in more detail.

Here, we focused on the (semi)­quantitative assessment of 25-hydroxyvitamin D_3_ in serum by acquiring data in the multiple reaction (MRM) mode, as routinely performed for vitamin D metabolite measurements (Figure 7). The deviation of measured concentrations between *in stillo* and *in vitro* analyses was 15.4%, which was deemed reasonable considering the proof-of-concept nature of the experimental levitation setup. Using stable isotope standards for internal calibration is expected to lead to improved accuracy and precision and much closer agreement between the two approaches. The *in stillo* approach exhibited a relative standard deviation of 5.9% (*n* = 3) within *in stillo* measurements, while the *in vitro* analysis was slightly more precise at 4.0% (*n* = 3). The slightly higher value may again be due to the manual steps required in the current levitator setup. The preliminary evaluation of the analytical figures of merit also yielded a limit of quantification (LOQ) of 2.9 ng/mL (*in stillo*) and 1.9 ng/mL (*in vitro*), respectively (Supporting Information, Experimental Methods). As human 25­(OH)­D levels are considered deficient at <20 ng/mL, insufficient at <30 ng/mL and adequate between 30–50 ng/mL, the achieved LOQ demonstrate the *in stillo* workflow’s principle fit-for-purpose performance. Furthermore, we believe that there is strong optimization potential for the *in stillo* analysis, which will likely provide similar or even better results than *in vitro* assay. The *in stillo* analysis drastically shortened the overall analysis time. The preparation time was reduced from approximately 90 min (*in vitro*) to 12 min, while lowering the required sample volume by approximately 90%. In terms of sample throughput, microvessels, such as microconical tubes or microplates, provide inherent advantages over conventional vials as parallelized, automated liquid handling systems can be implemented for liquid transfers. However, microvessels are prone to exhibiting surface adsorption of sample, high risk of contamination and reduced mass transfers. In contrast, acoustic levitation offers a containerless, highly efficient single-droplet processing platform with strongly reduced sample contamination, accelerated extraction kinetics and rapid derivatization rates. While the current manual proof-of-concept platform is limited in terms of parallel processing, future automation of injection, acoustic pressure control and ejection will increase sequential sample throughput and readily provide on-demand, freshly prepared samples. Future developments will integrate a digitally controlled syringe pump and an acoustic pressure regulator, enabling automated control of the sequencing workflow via a microcontroller unit (e.g., Arduino or ESP32)

**Figure 7:**
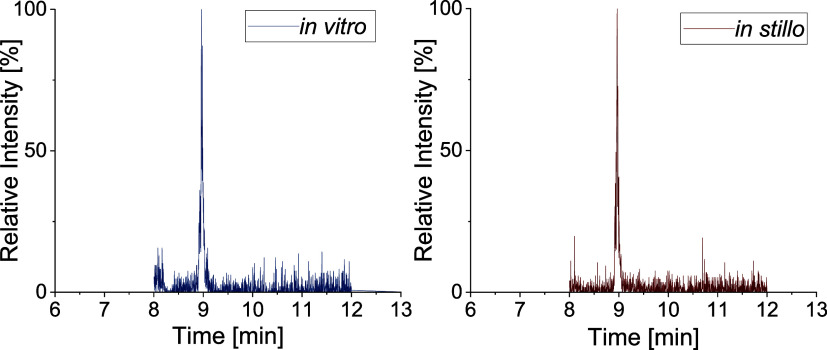
MRM chromatogram (*m*/*z* 558 → 298) for 25­(OH)­D_3_ (*c* = 25.4 ng/mL) of a human serum sample; *in vitro* (left, blue) and *in stillo* (right, red).

## Conclusions

In this proof-of-concept study, we demonstrated the potential of acoustic levitation for integration into a complete sample preparation workflow for 25-hydroxyvitamin D_3_ in human serum. We demonstrated a contactless droplet handling technique via field modulation and spatial displacement for merging and reseparation of two immiscible solvents (aqueous serum and extraction solvent). We were able to perform *in stillo* chemical derivatization of 25­(OH)­D_3_ at strongly enhanced reaction rates under the chosen conditions as well as liquid–liquid extractions in a single levitated droplet. The required sample size was reduced by 90% compared to the standard vitamin D assay and the enhanced reaction rates combined with rapid droplet extraction contributed to much faster sample processing at the same time.

The presented *in stillo* workflow demonstrated very promising results, suggesting its utilization in automated sample processing workflows after further optimization steps. Areas for improvement concern the controlled handling of the levitated droplets, to prevent occasional fragmentation or combustion, in particular when very volatile extractant solvents are used. Also, analyte losses can still occur in the described manual offline setup. Automation of sample handling will address these challenges in the future, as well as direct measurements of the analytes from the levitated droplet, as previously demonstrated by us for direct mass spectrometric interrogation of analytes from acoustically levitated droplets.^19,24,53^ We will further automate the method by computer-controlling many of the described manual field control steps and by including more complex sample preparation procedures, e.g., robotic sample introduction, to further increase sample throughput and remove manual handling steps. A fully automated approach will also allow us to use even smaller sample sizes, e.g., volumes <1 μL. Further fundamental levitation-based studies regarding the role of matrix viscosity, matrix effects and detection of other analyte targets will be addressed.

## Supplementary Material



## Notes

Chemicals and materials; detailed experimental levitation methods; standard preparation; sample derivatization; serum protein precipitation; LOD and LOQ calculations; and supporting levitation figures and mass spectra (PDF)

## Abbreviations

LC-MS/MS: liquid chromatography-tandem mass spectrometry
FA: formic acid
MRM: multiple reaction monitoring
PTAD: 4-phenyl-1,2,4-triazolin-3,5-dion
TIC: total ion current
*t*_*R*_: retention time
UV/vis: ultraviolet–visible
XRD: X-ray diffraction
