# Supplementary material for: Lab-in-a-Droplet: Utilizing Acoustic Levitation for Miniaturized Sample Preparation and LC-MS/MS, Exemplified by Measurement of Vitamin D in Serum
Source: Anal Chem. 2026 Jul 22;98(30):22034–42. doi: 10.1021/acs.analchem.6c03465 (PMC13449226; doi:10.1021/acs.analchem.6c03465)
Supplement: Supplementary file 1 [file ac6c03465_si_001.pdf]

Supporting Information

Lab-in-a-droplet: utilizing acoustic levitation for miniaturized sample preparation and LC-MS/MS, exemplified by measurement of vitamin D in serum

Geron Ratzek and Dietrich A. Volmer*

Department of Chemistry, Humboldt-Universität zu Berlin, Brook-Taylor-Straße 2, Berlin 12489, Germany

Table of Contents

Experimental Methods 2

Supplementary Figures and Data...4

Funding source

We are grateful to the German Research Foundation (DFG VO 1355/10-1) for supporting this project.

*Corresponding author:

Prof. Dr. Dietrich Volmer
Humboldt University Berlin
Department of Chemistry
12489 Berlin, Germany

Tel +49 30 2093 7588
Email: Dietrich.Volmer@hu-berlin.de

Experimental Methods

Levitation and sample preparation were performed under ambient conditions at room temperature (20-25 °C).

Chemicals and materials

All chemicals used in this study were purchased from TCI (Zwijndrecht, Belgium), Merck (Darmstadt, Germany), and Sigma-Aldrich (St. Gallen, Switzerland). Pooled human serum samples were obtained from Merck; UHPLC-MS-grade acetonitrile and methanol from Chemsolute (Renningen, Germany), formic acid (97%) from AlfaAesar (Karlsruhe, Germany). Ultrapure water was generated by a Millipore Direct-Q8 purification system (Bedford, MA, USA).

Spatial Manipulation of Miscible and Immiscible Solvents

The initial distance between the transducer and levitator was set to 2.58 cm. Five µL of the first solvent was injected into the central low-pressure point of the acoustic field. The acoustic sound pressure was adjusted by gradually modulating the power source's output voltage until stable levitation without oscillation was achieved. Afterward, the second solvent (5 µL) was injected into an adjacent low-pressure nodal point. Through rapid manual modulation of the acoustic sound pressure (ΔHF = 0.4-0.7 W), contactless spatial manipulation of the droplets was achieved. The explicit sound-pressure adjustment depended on the physicochemical properties of the solvents and was tested before further experiments were performed (**Table S1**).

Subsequently, the resulting single- or double-phase droplet was fragmented (single-phase) or separated (double-phase) by rapid modulation of the acoustic sound pressure in the specified range.

Gradual modulation of the acoustic sound pressure provided no ability for spatial manipulation and resulted in droplet vibration, atomization (increasing sound pressure) or loss of levitation (decreasing sound pressure).

Acoustic streaming and oscillations

Ten µL of 1-octanol (dyed with methyl red) was injected into the central nodal point of the acoustic field. The acoustic pressure was adjusted until stable levitation was achieved. Subsequently, the sound pressure was slowly adjusted (ΔHF = 0.2 W). Acoustic streaming was observed through undissolved methyl red, which followed the laminar flow within the levitating droplet. Droplet oscillation was observed after the initial distance was gradually modulated (Δx = 2 mm). Reverting to the initial conditions resulted in the absence of acoustic streaming or oscillation.

Acoustic levitation solvent exchange

Five µL of a volatile solvent (e.g., cyclohexane or ethyl acetate) was injected into the central nodal point of the acoustic field. Upon stabilization, the droplet was left to evaporate to near dryness under atmospheric conditions. Subsequently, 5 µL of fresh solvent (e.g., water or methanol) was

injected into the same nodal point. After 30 s, no residual initial solvent was observed on the surface of the fresh solvent.

Standard preparation, derivatization, and calibration

Standards of 25-hydroxyvitamin D_3 (25(OH)D) and PTAD were dissolved in dry acetonitrile to yield 40 ng/mL and 200 ng/mL stock solutions and were stored at -20 °C. Working solutions were obtained by serial acetonitrile dilution before analysis. Subsequently, 5 µL 25(OH)D standard was injected into the central nodal point and PTAD standard (5 µL) was added. The droplet was left to react within the acoustic field for 3 min. Afterward, the droplet was manually collected and quenched with 30 µL methanol. The solvents were evaporated under reduced pressure, and the residues were reconstituted in 100 µL of MeOH/H_2O (85/15 v/v) to reach the corresponding final concentrations of 0.5, 1.0, 1.5, 2.0, 2.5 and 3.0 ng/mL. The resulting standards were submitted to LC-MS/MS and the corresponding peak areas were quantified. Standards were measured in triplicate. Corresponding *in vitro* standards were prepared in accordance with previously published literature. [1, 2]

Serum protein precipitation

Five µL of human blood serum were injected into the central nodal point. The acoustic field pressure was adjusted until stable levitation without acoustic streaming was achieved. Afterward, dry acetonitrile (5 µL) was added to the levitating droplet. Immediate protein precipitation was observed by forming a yellowish-white solid within the suspended droplet. Gradual agglomeration was achieved by gradually modulating the acoustic sound pressure (ΔHF = 0.2 W). Subsequently, the agglomerate was collected at the bottom of the droplet by slowly decreasing the acoustic sound pressure (ΔHF = -0.6 W). Distance adjustments and resulting oscillation led to rotational movement of the agglomerated precipitate around the z-axis. Attempts to separate the solid with the contactless manipulation approach yielded no distinct separation.

Limit of detection and limit of quantification

The instrumental limit of detection (LOD) and the limit of quantification (LOQ) were evaluated through the linear regression method of a six-point calibration curve analyzed in triplicate (n=3). The metrics were defined as $3 \cdot \sigma/m$ and $10 \cdot \sigma/m$, respectively, where σ is the sample standard deviation of the lowest concentration point and m is the slope of the regression line. The regression equation yielded a coefficient of determination (R^2) of 0.997, establishing LOD of 0.007 ng/mL and LOQ of 0.022 ng/mL. The method LOD and LOQ were determined based on signal-to-noise ratios (S/N) of 3 and 10, respectively. Peak-to-peak noise (N) was determined from the chromatogram's signal-free baseline interval (9.1-10.5 min) adjacent to the analyte peak, exhibiting S/N=16.7 at 4.0 ng/mL for the *in stillo* serum sample and S/N=20.0 at 3.8 ng/mL for the *in vitro* sample. Consequently, the corresponding method LOQs were determined to be 2.9 ng/mL (*in stillo*) and 1.9 ng/mL (*in vitro*), respectively.

Supporting Figures and Data

Table S1: Levitation stability test of different solvents and solvent pairs inside the acoustic field. Solvents that are too volatile could only be levitated at low *HF* values < 3 W, as otherwise, atomization occurred.

Levitated solvents	Levitated but too volatile	Levitated solvent pairs
H_2O	DCM	Can be modulated
1-Octanol / 2-Octoanol	Trichloromethane	H_2O / MeOH
Methanol	Et_2O	H_2O / EtOH
Ethanol		H_2O / Cyclohexane
*(tert-)*Butanol		H_2O / 1-Octanol (2-Octanol)
Hexanol		MeOH / Cyclohexane
Acetonitrile		ACN / Cyclohexane
Dimethyl formamide		ACN / Ethyl acetate
Ethyl acetate		Cannot be modulated
Hexane / Cyclohexane		H_2O / DCM
Octane		H_2O / Et_2O

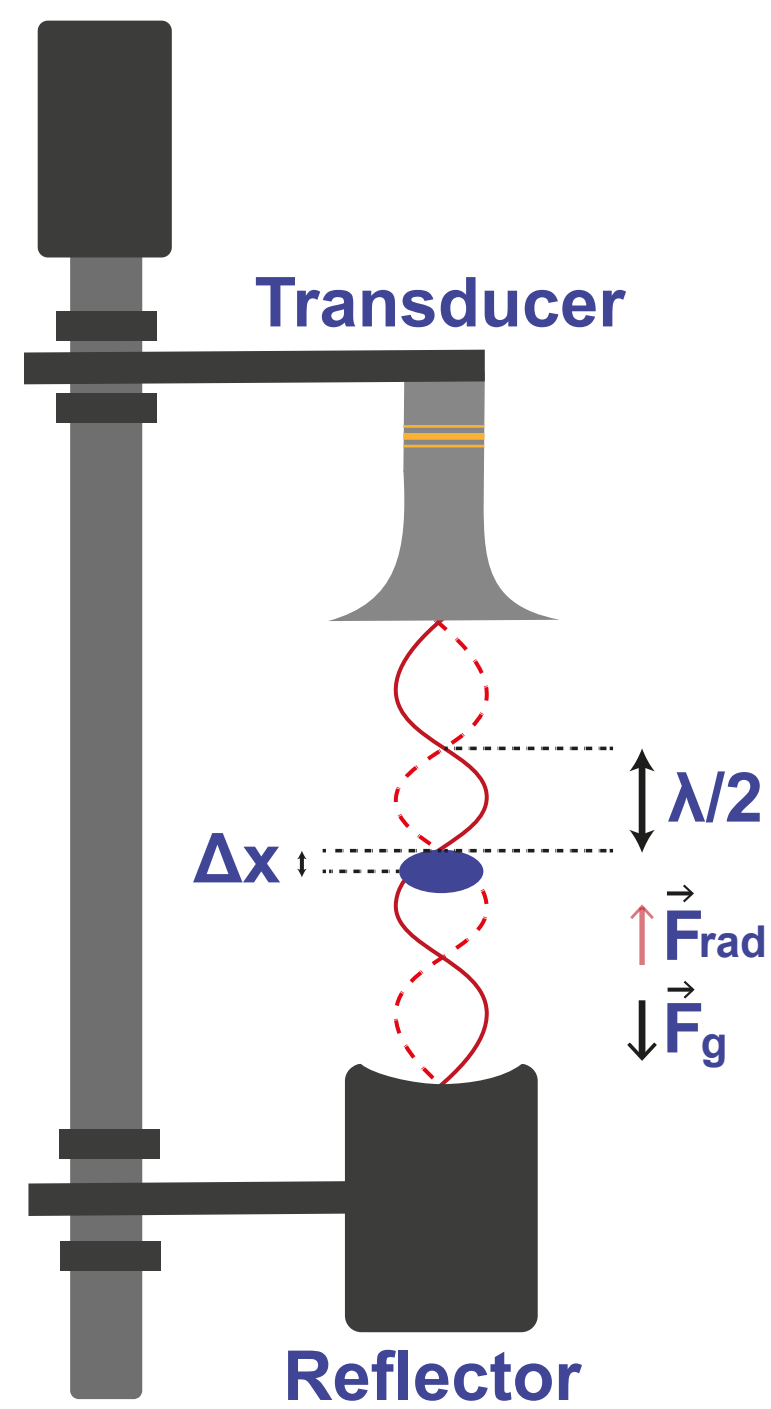

Figure S1: Schematic representation of the mode of action of acoustic levitation, with $\lambda/2$ = distance of low-pressure nodal points, Δx = offset of theoretical and actual levitation node, F_{rad} = radiational force and F_g = gravitational force.

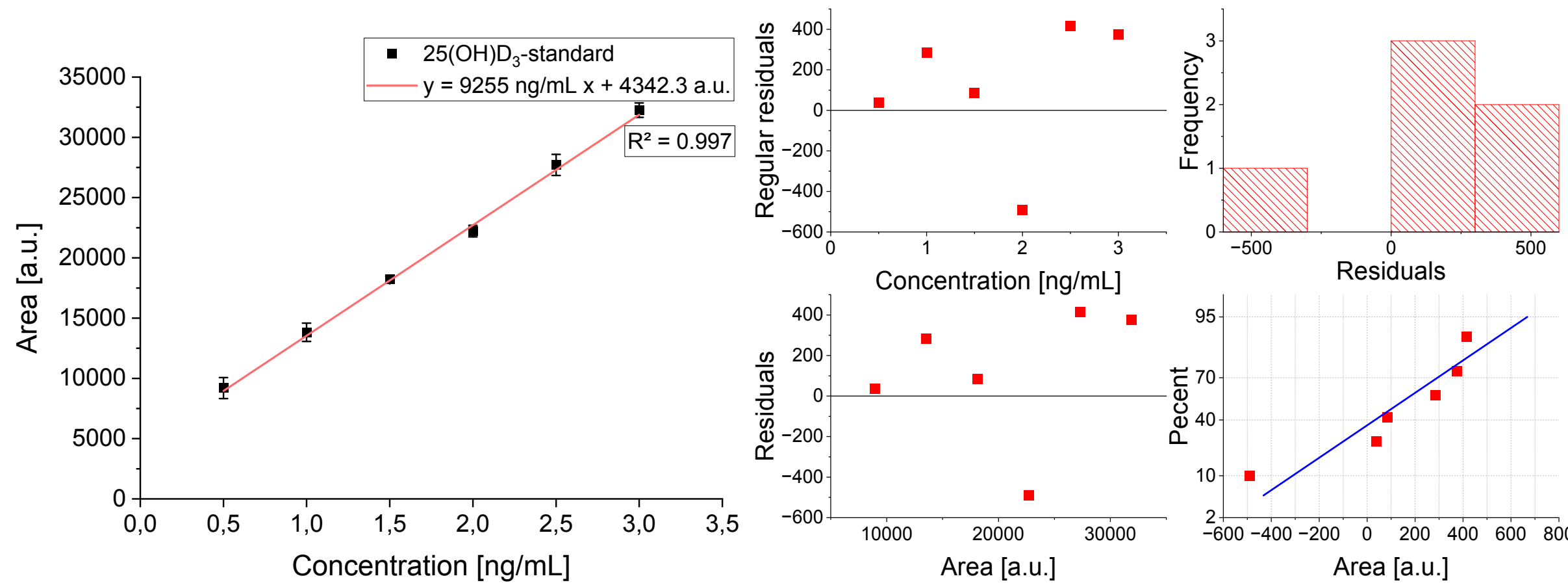

Figure S2: Calibration curve for measurement of 25-hydroxyvitamin D_3 in human serum after liquid-liquid extraction using acoustic levitation (left) with corresponding residual plots (right).

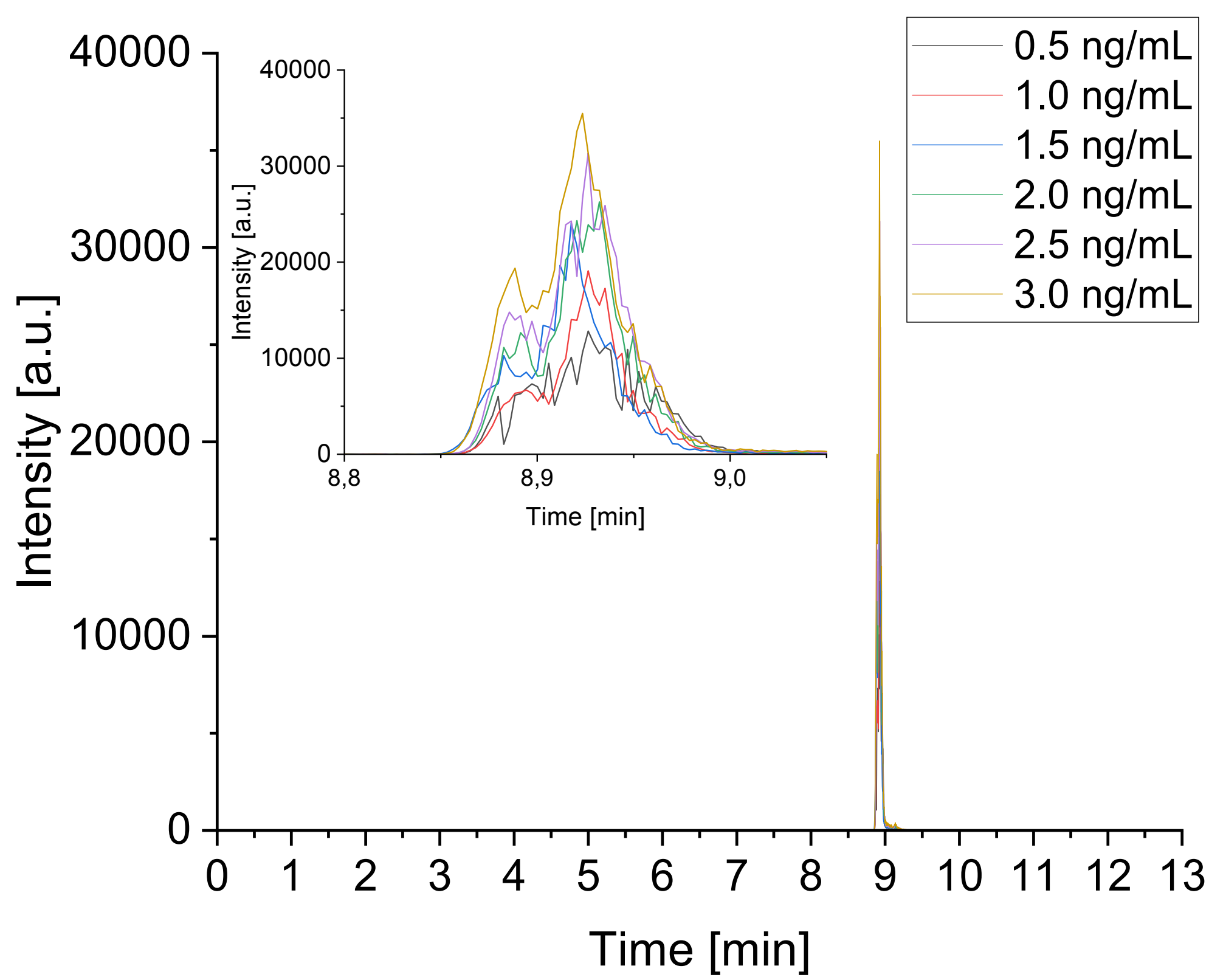

Figure S3: MRM chromatograms (*m/z* 558 → 298) for 25-hydroxyvitamin D_3 solvent standards at various concentrations (0.5, 1.0, 1.5, 2.0, 2.5, 3.0 ng/mL) to bracket the target range of 1.0-2.5 ng/mL after reconstitution and dilution of the human serum sample.

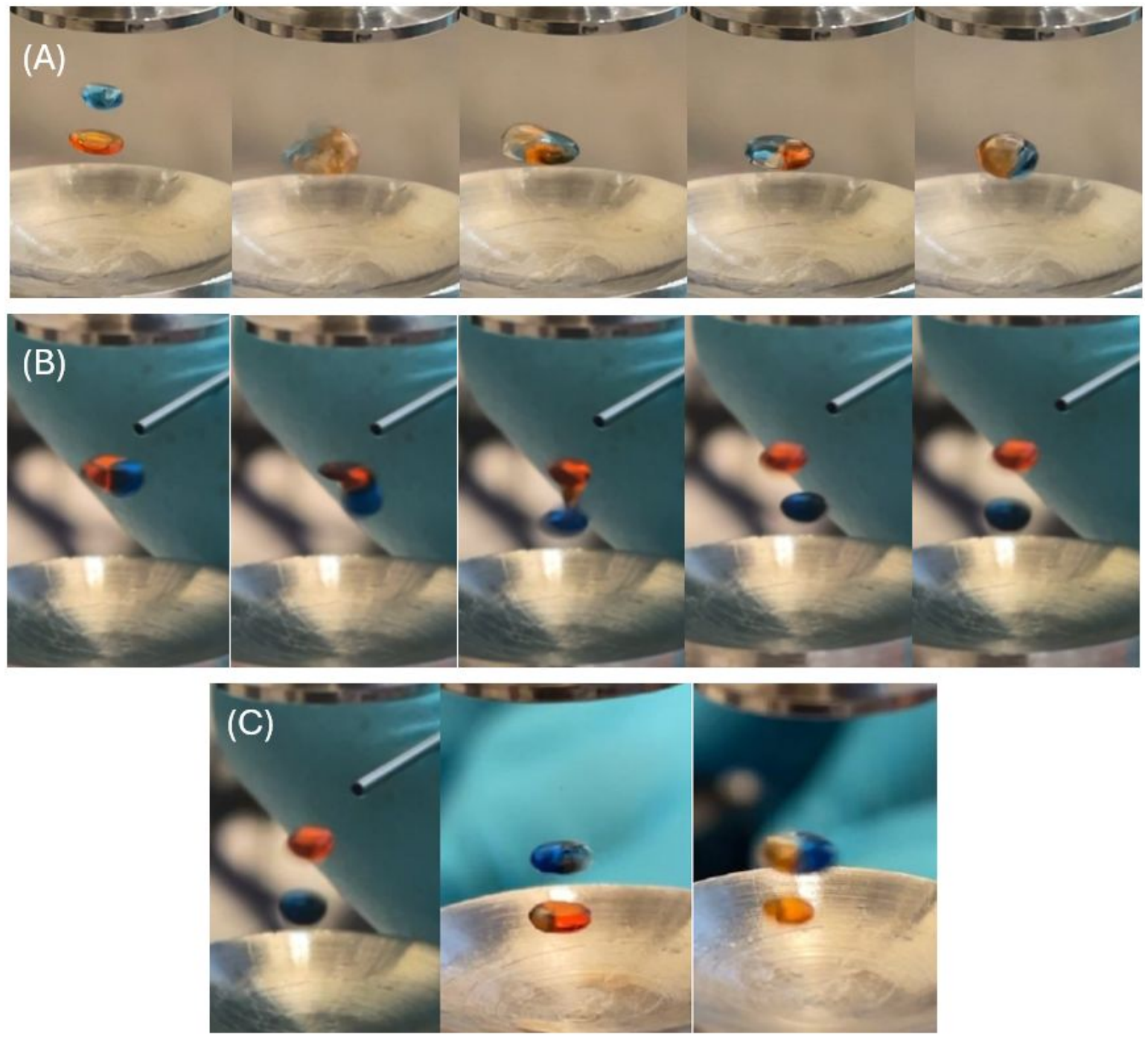

Figure S4: (A) Merging of 5 µL droplets of two immiscible solvents (water, dyed blue with indigo carmine; 1-octanol, dyed orange with methyl red). (B) Separation of the previously merged droplets. (C) Different separation patterns; full separation (left), remaining residual solvent attached to isolated droplet (middle), partial fragmentation (right).

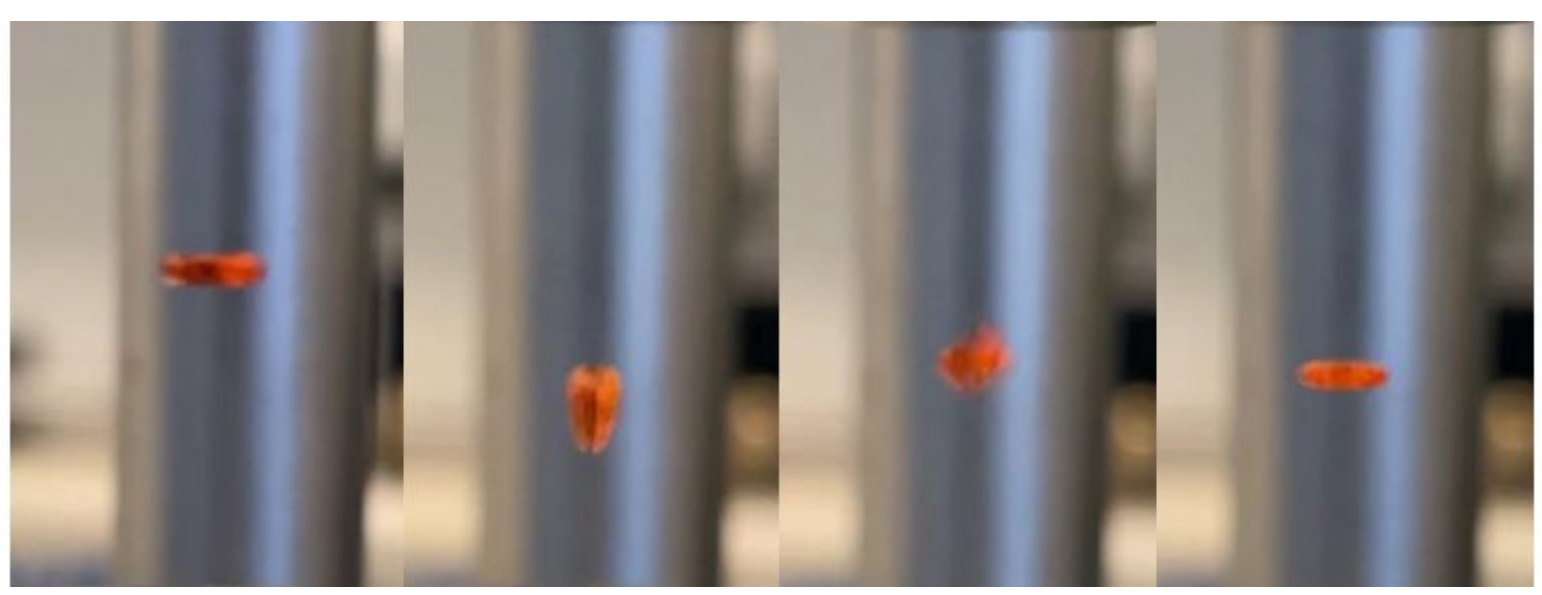

Figure S5: Spatial manipulation of a single water droplet (dyed with methyl orange) within a single-axis levitator.

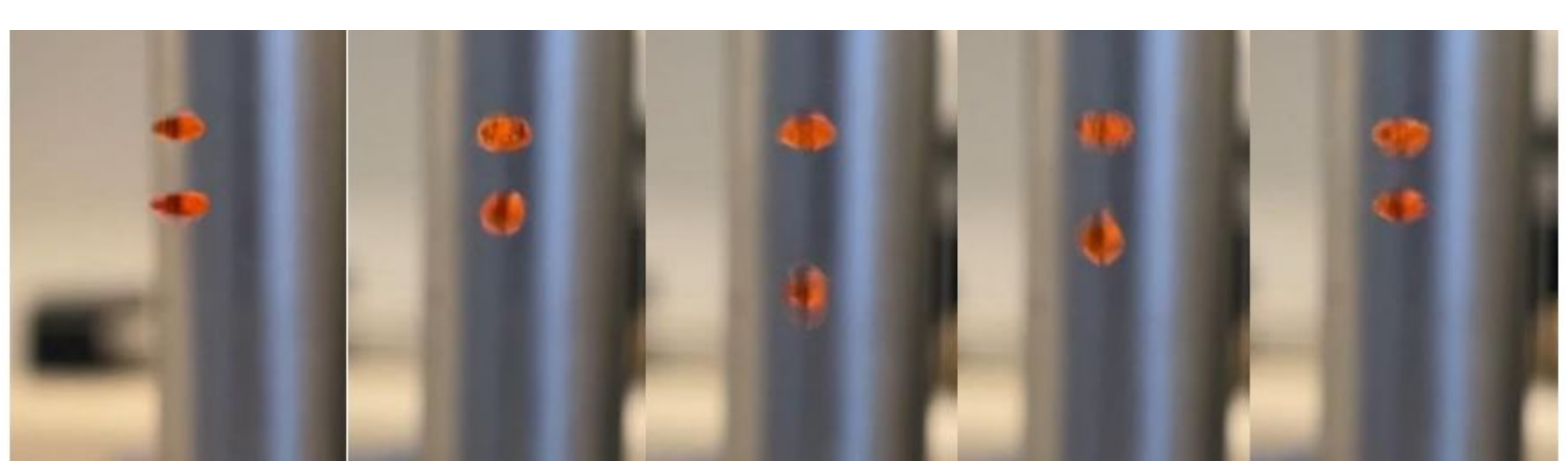

Figure S6: Spatial manipulation of the lower water droplet (dyed with methyl orange) within a single-axis levitator. Successive spatial manipulation was achieved by rapid modulation of the acoustic sound pressure. We attribute this behavior to the strong sound pressure of the central low-pressure point, which exerts more force on the central droplet.

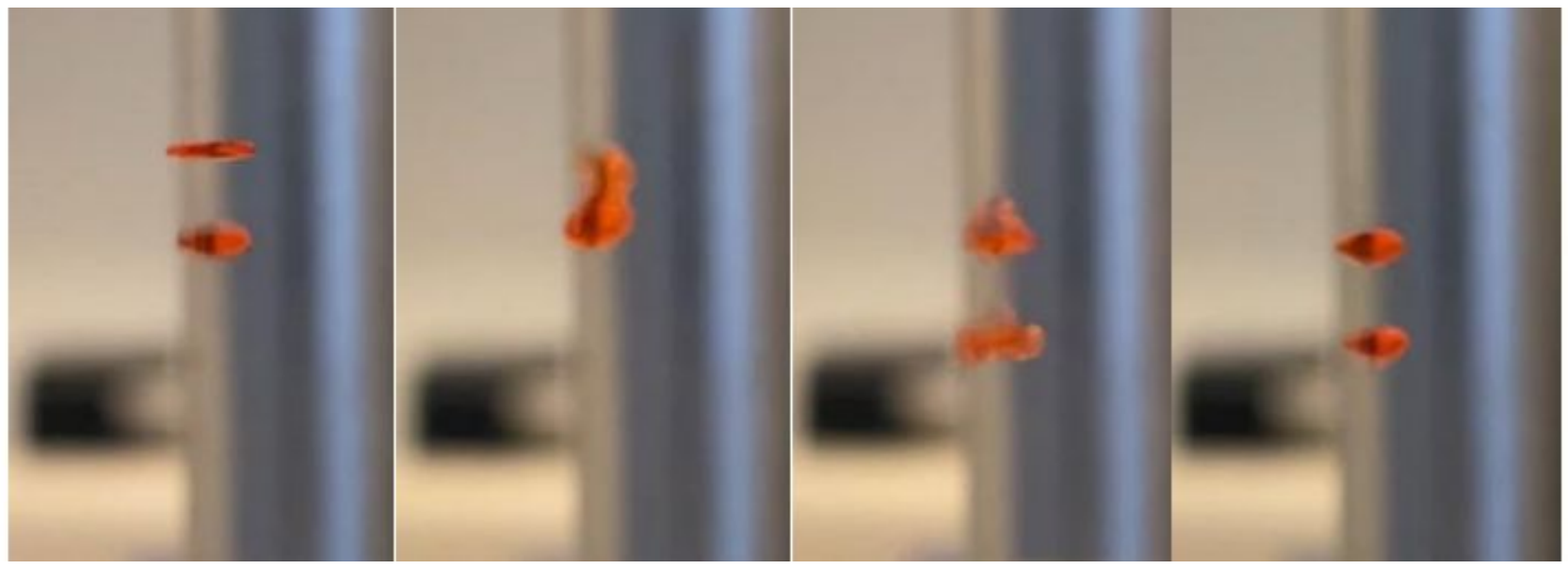

Figure S7: Spatial manipulation of two water droplets (dyed orange with methyl orange) within a single-axis levitator. Partial mixing was observed during spatial transition due to excessive vibration (uncontrolled modulation) during spatial manipulation.

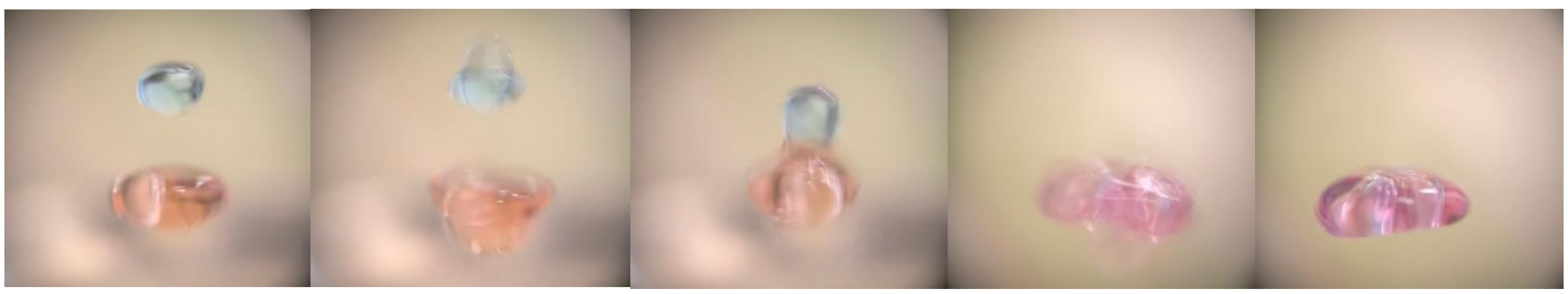

Figure S8: Spatial manipulation of two miscible solvents, water (upper, dyed blue with indigo carmine) and methanol (lower, dyed orange with methyl red).

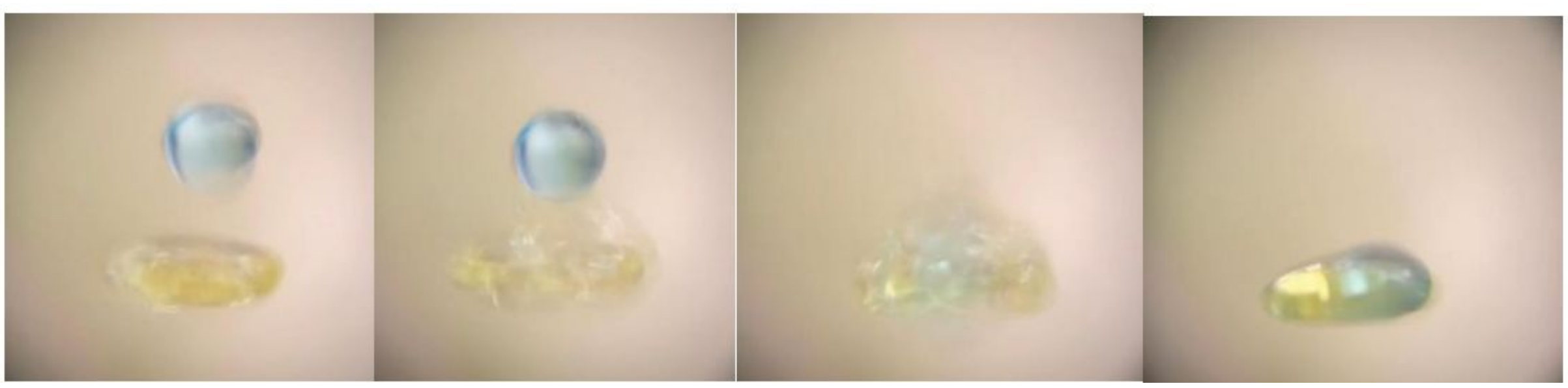

Figure S9: Spatial manipulation of two immiscible solvents, acetonitrile (top, dyed blue with indigo carmine) and cyclohexane (bottom, dyed yellow with methyl red).

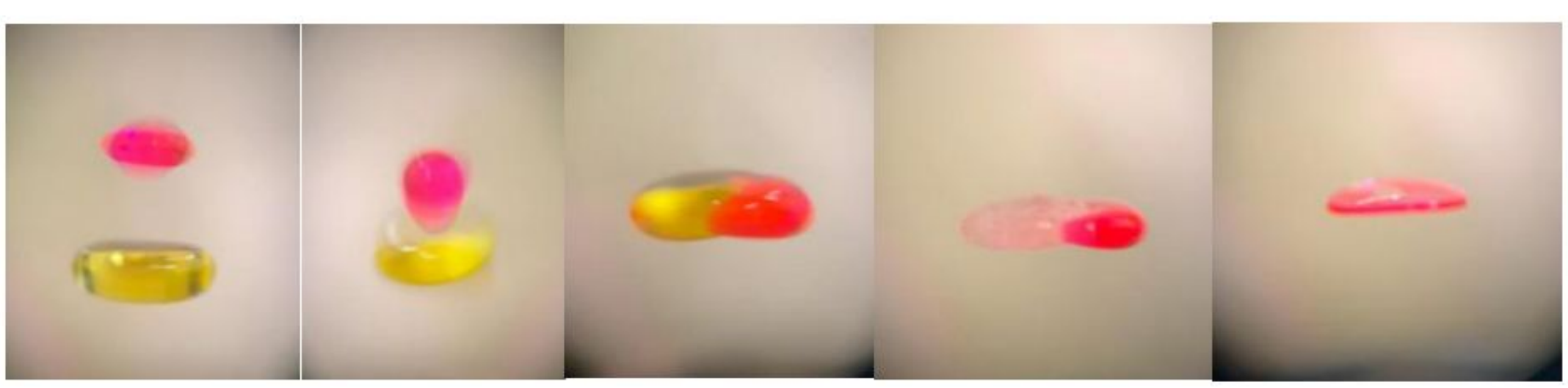

Figure S10: Spatial manipulation of two immiscible solvents, methanol (top, dyed red with rhodamine-B) and cyclohexane (bottom, dyed yellow with methyl red). After merging, the formation of a viscous layer on the cyclohexane surface and discoloration was observed.

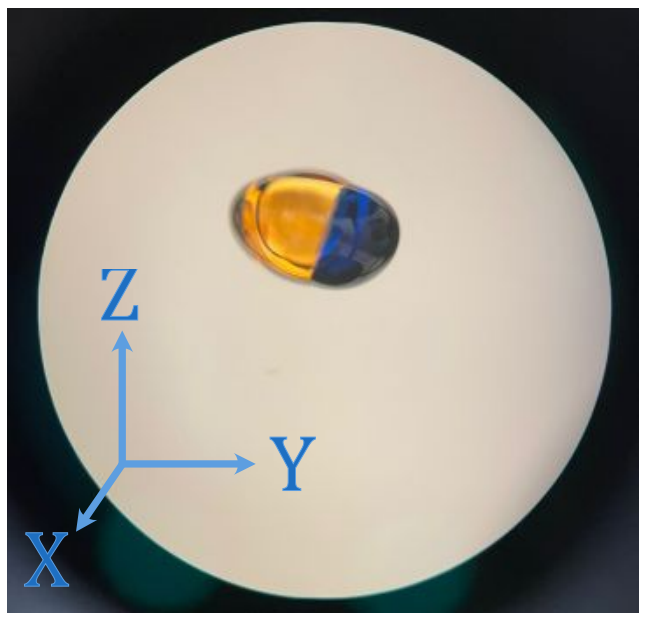

Figure S11: Interfacial phase plane observed in immiscible solvent merging, water (right, dyed blue with indigo carmine) and 1-octanol (left, dyed orange with methyl red). Due to the acoustic sound field, the droplet consistently adopted a specific orientation (along the *yz*-plane).

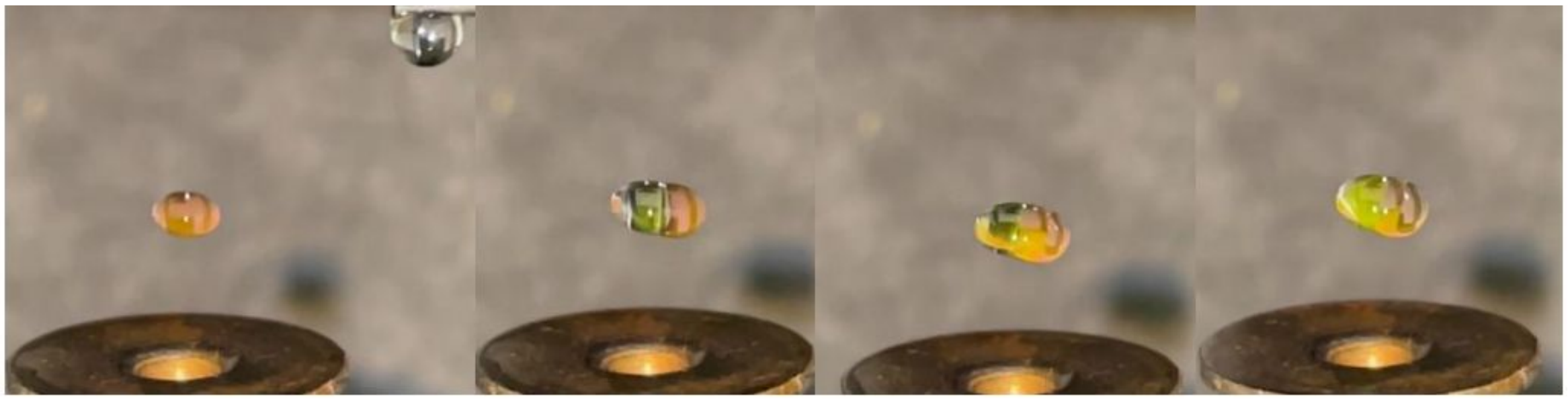

Figure S12: Visual extraction of rhodamine-B from 1-octanol (pinkish, orange) into water (fluorescent yellow).

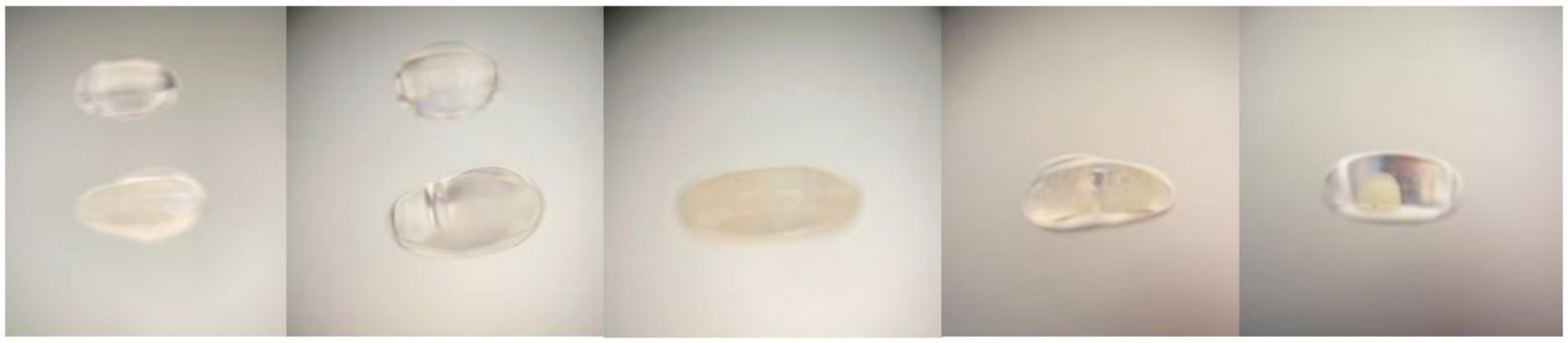

Figure S13: Merging of acetonitrile (upper droplet) with human serum (lower droplet) and subsequent precipitation and agglomeration of serum proteins.

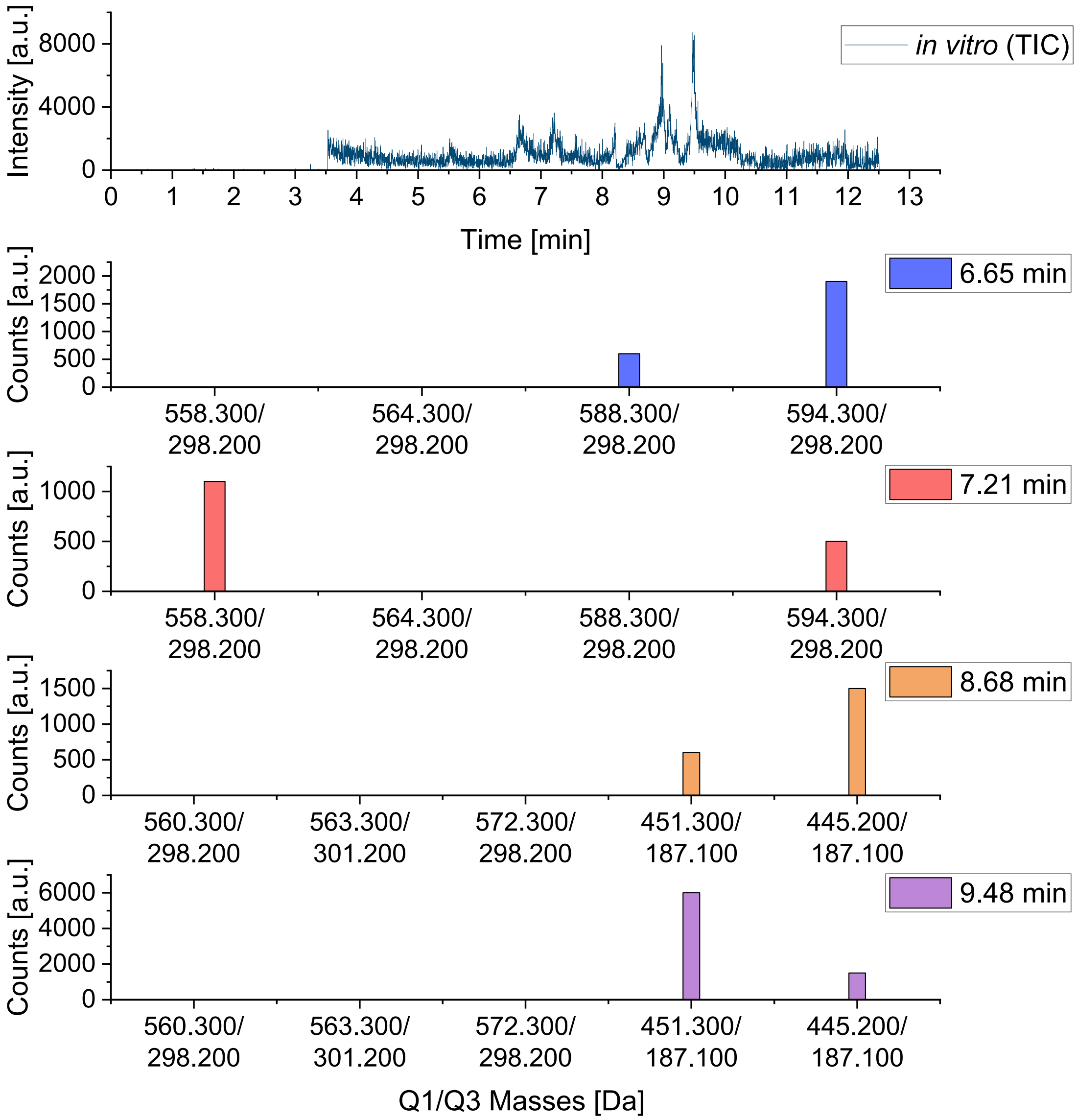

Figure S14: Total ion current (TIC)-chromatogram of the *in vitro* sample (top trace, blue) and the detected analytes MRM transitions (blue, red, beige, purple).

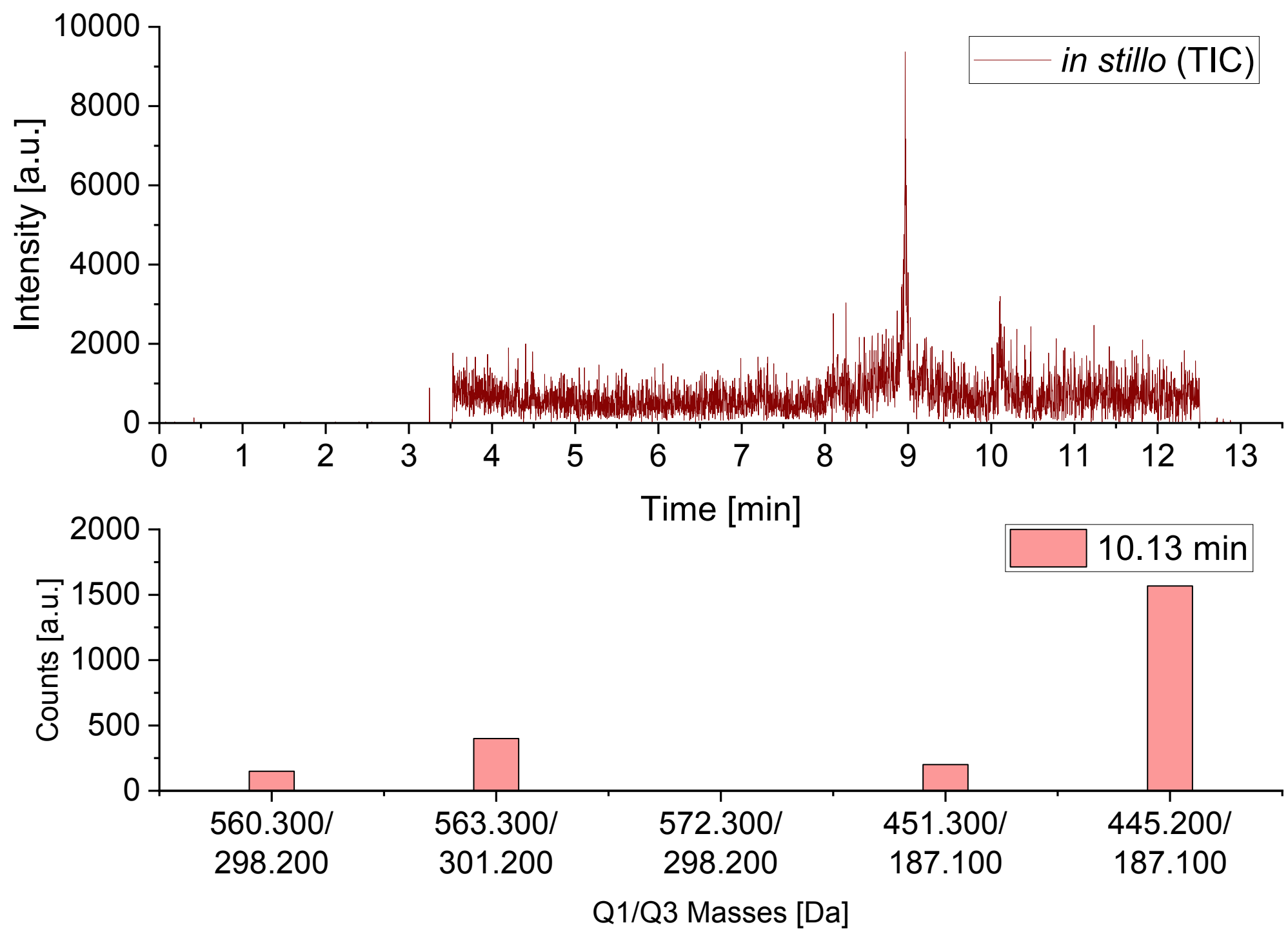

Figure S15: Total ion current (TIC)-chromatogram of the *in stillo* sample (top trace, red) and the MRM transitions at 10.13 min (bottom, red).

References

(1) Ding, S.; Schoenmakers, I.; Jones, K.; Koulman, A.; Prentice, A.; Volmer, D. A. Quantitative determination of vitamin D metabolites in plasma using UHPLC-MS/MS. *Anal Bioanal Chem* **2010**, *398* (2), 779-789. DOI: 10.1007/s00216-010-3993-0 From NLM.

(2) Alexandridou, A.; Schorr, P.; Volmer, D. A. Comparing derivatization reagents for quantitative LC–MS/MS analysis of a variety of vitamin D metabolites. *Analytical and Bioanalytical Chemistry* **2023**, *415* (19), 4689-4701. DOI: 10.1007/s00216-023-04753-0.
